# Analysis of a SARIMA-XGBoost model for hand, foot, and mouth disease in Xinjiang, China

**DOI:** 10.1017/S0950268823001905

**Published:** 2023-12-04

**Authors:** Haojie Man, Hanting Huang, Zhuangyan Qin, Zhiming Li

**Affiliations:** 1 School of Mathematics and Statistics, Beijing Institute of Technology, Beijing, China; 2 School of Mathematical Sciences, Beihang University, Beijing, China; 3 College of Mathematics and System Science, Xinjiang University, Urumqi, China

**Keywords:** hand, foot, and mouth disease, SARIMA model, XGBoost algorithm, GTWR model

## Abstract

Hand, foot, and mouth disease (HFMD) is a common childhood infectious disease. The incidence of HFMD has a pronounced seasonal tendency and is closely related to meteorological factors such as temperature, rainfall, and wind speed. In this paper, we propose a combined SARIMA-XGBoost model to improve the prediction accuracy of HFMD in 15 regions of Xinjiang, China. The SARIMA model is used for seasonal trends, and the XGBoost algorithm is applied for the nonlinear effects of meteorological factors. The geographical and temporal weighted regression model is designed to analyze the influence of meteorological factors from temporal and spatial perspectives. The analysis results show that the HFMD exhibits seasonal characteristics, peaking from May to August each year, and the HFMD incidence has significant spatial heterogeneity. The meteorological factors affecting the spread of HFMD vary among regions. Temperature and daylight significantly impact the transmission of the disease in most areas. Based on the verification experiment of forecasting, the proposed SARIMA-XGBoost model is superior to other models in accuracy, especially in regions with a high incidence of HFMD.

## Introduction

Hand, foot, and mouth disease (HFMD) is a common infectious disease caused by a group of enteroviruses, such as Coxsackie virus A16 and Enterovirus 71 [1]. This disease is mainly transmitted through person-to-person contact and respiratory droplets. The main manifestations are fever, skin eruptions on hands and feet, and vesicles in the mouth [2]. The disease is characterized by rapid progression. Once respiratory complications such as pulmonary oedema and pulmonary haemorrhage occur, patients may die quickly [3]. From 2009 to 2018, more than 70000 HFMD cases were reported in Xinjiang, including 98 severe cases and 11 fatal cases [4]. Given the severity and fatality rates, it is vital to analyze the characteristics and factors influencing the prevention of HFMD transmission in Xinjiang.

According to the transmission mechanism of infectious diseases, meteorological conditions may influence the incidence, the transmission range, and the susceptibility of the population to diseases [5–7]. Various studies have shown that meteorological factors such as temperature, rainfall, humidity, air pressure, light, and wind speed are tightly associated with HFMD [8–11]. In addition, the meteorological factors demonstrated significant spatial and temporal variation in HFMD incidence, and they revealed a nonlinear correlation with the incidence [12, 13]. These works demonstrate the importance of analyzing meteorological factors in predicting HFMD. Since the HFMD incidence has prominent seasonal characteristics [14–16], the seasonal autoregressive integrated moving average (SARIMA) model has been widely used in predicting seasonal infectious diseases for its efficient forecasting ability for periodic time series. Many studies have been conducted using the SARIMA model to predict HFMD incidence [17–20]. In practice, the time series of HFMD often contain linear and nonlinear patterns. However, the SARIMA model is limited by its linear assumptions and cannot capture the nonlinear patterns [21]. To capture the nonlinear correlations between patient numbers and meteorological factors, machine learning algorithms have shown significant advantages over traditional statistical models [22]. Therefore, many machine learning methods have been applied to predict the number of HFMD cases, such as long- and short-term memory networks [23, 24], random forest [25], recurrent neural network [26], and support vector regression [27]. However, past studies have not considered the influence of meteorological factors on both time and space, rendering most models non-generalizable to specific locations or times.

From the above analysis, it is clear that machine learning models can compensate for the shortcomings of the SARIMA model, specifically its inability to address nonlinearity between the number of infected people and the influencing factors. In contrast, machine learning models, while pursuing higher prediction accuracy, are prone to overfitting, which can undermine the credibility of their predictions [28]. However, most current research focused only on either the seasonal characteristics of transmission or the correlation between transmission and meteorological factors when making predictions. Consequently, a meaningful proposition is whether we can integrate a traditional time series model with a machine learning algorithm to build a combined model with a higher prediction accuracy and better generalization ability.

This paper proposes a SARIMA-XGBoost combined model for predicting HFMD time series. The SARIMA model is used to capture the seasonal trends in the disease, while the XGBoost (eXtreme Gradient Boosting) algorithm is applied to account for the effects of meteorological factors on transmission. The geographically and temporally weighted regression (GTWR) model analyzes the impact of meteorological factors from both temporal and spatial perspectives. The effectiveness of the combined model is investigated and validated. The remainder of the paper is organized as follows. Section ‘Data source and factor analysis’ presents the data source and factor analysis. Section ‘The SARIMA-XGBoost model’ introduces various components of the model, i.e. the SARIMA model and the XGBoost algorithm, followed by the construction method of the combined model. Section ‘Experiment and analysis’ presents experiments that analyze the results and compare them with other models to validate the proposed method. Finally, Section ‘Discussion and Conclusion’ contains the concluding remarks and outlines future research directions.

## Data source and factor analysis

### Data source

Xinjiang Autonomous Region consists of five regions (Altay, Tarbagatay, Kashgar, Aksu, and Hotan), five autonomous prefectures (Ili, Bortala, Changji, Kizilsu, and Bayingol), and five cities (Urumqi, Karamay, Shihezi, Hami, and Turpan). The data on HFMD cases from 2008 to 2018 were sourced from [10]. The meteorological data, including monthly average temperature, average precipitation, barometric pressure, sunshine hours, average humidity, and wind speed, are sourced from NASA (https://ladsweb.modaps.eosdis.nasa.gov).

### Analysis of HFMD in 15 regions

**Figure 1.**
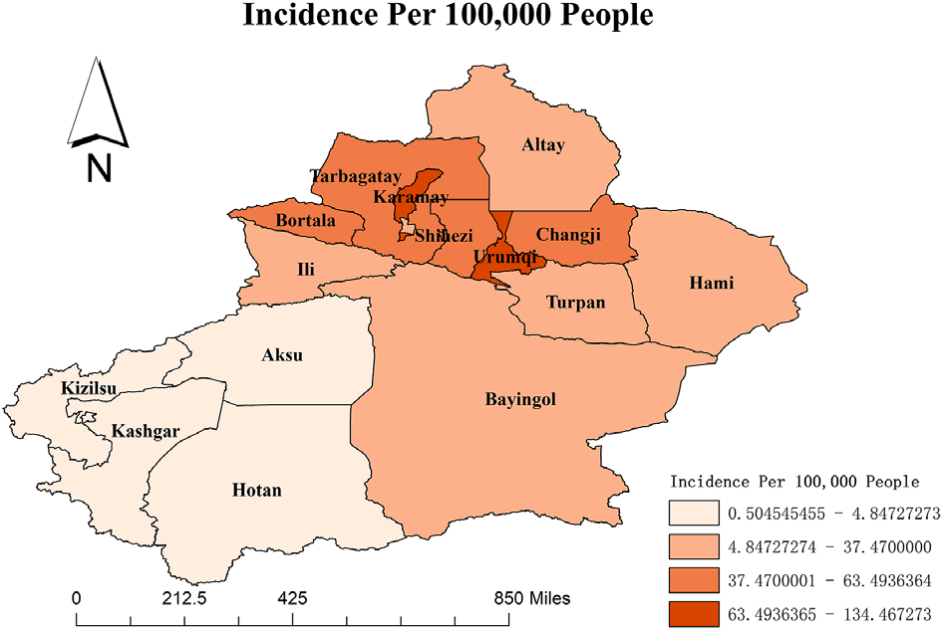
The HFMD incidence in various regions of Xinjiang province.

The average HFMD incidence is 38.84 per 100000 in the Xinjiang Autonomous Region from 2008 to 2018. Hereafter, all the following incidences are shown per 100000 population. The map in Figure 1 shows that the annual average HFMD incidence widely varied among different regions and is generated by the software ArcGIS 10.2 from http://eol.jsc.nasa.gov/ SearchPhotos/. The top two regions are Karamay and Urumqi, with incidence rates of 134.47 and 97.34, respectively. Hotan (0.50), Kashgar (0.67), Kizilsu (1.22), and Aksu (4.85) have relatively lower incidences.

**Figure 2.**
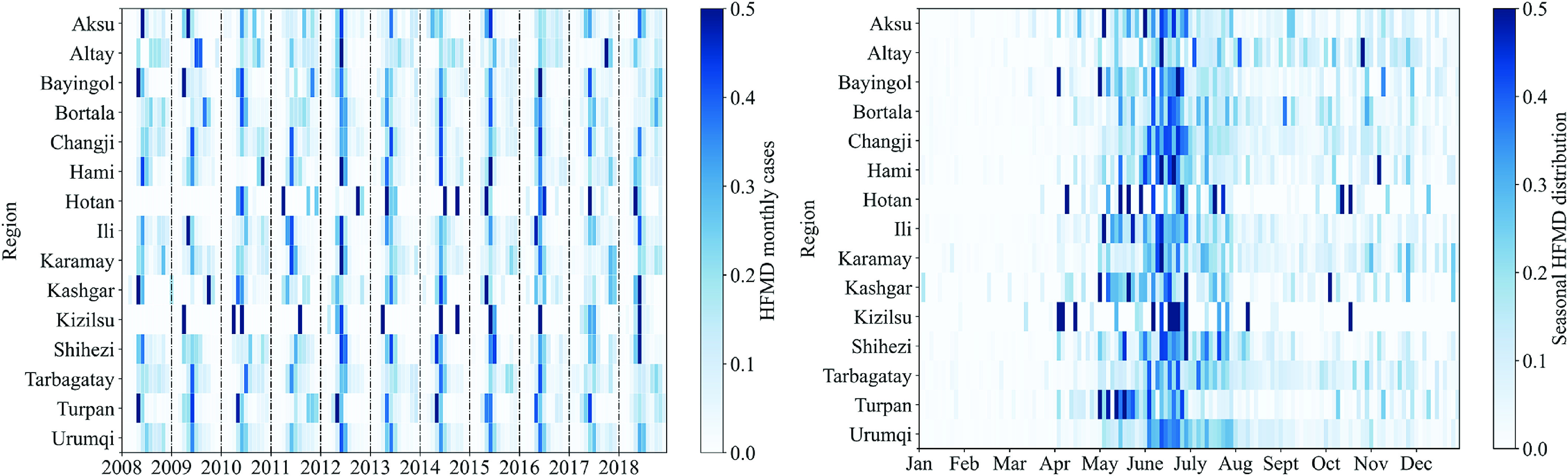
The HFMD cases by the month of illness onset, standardized by the number of annual cases.

Based on the monthly reported data on HFMD cases, Figure 2 reflects the incidences of HFMD in 15 regions from 2008 to 2018. Further, the prevalence of HFMD has prominent seasonal characteristics in Xinjiang. It is concentrated between May and August, reaching 77.54% of the total cases.

### Analysis of meteorological factors

**Figure 3.**
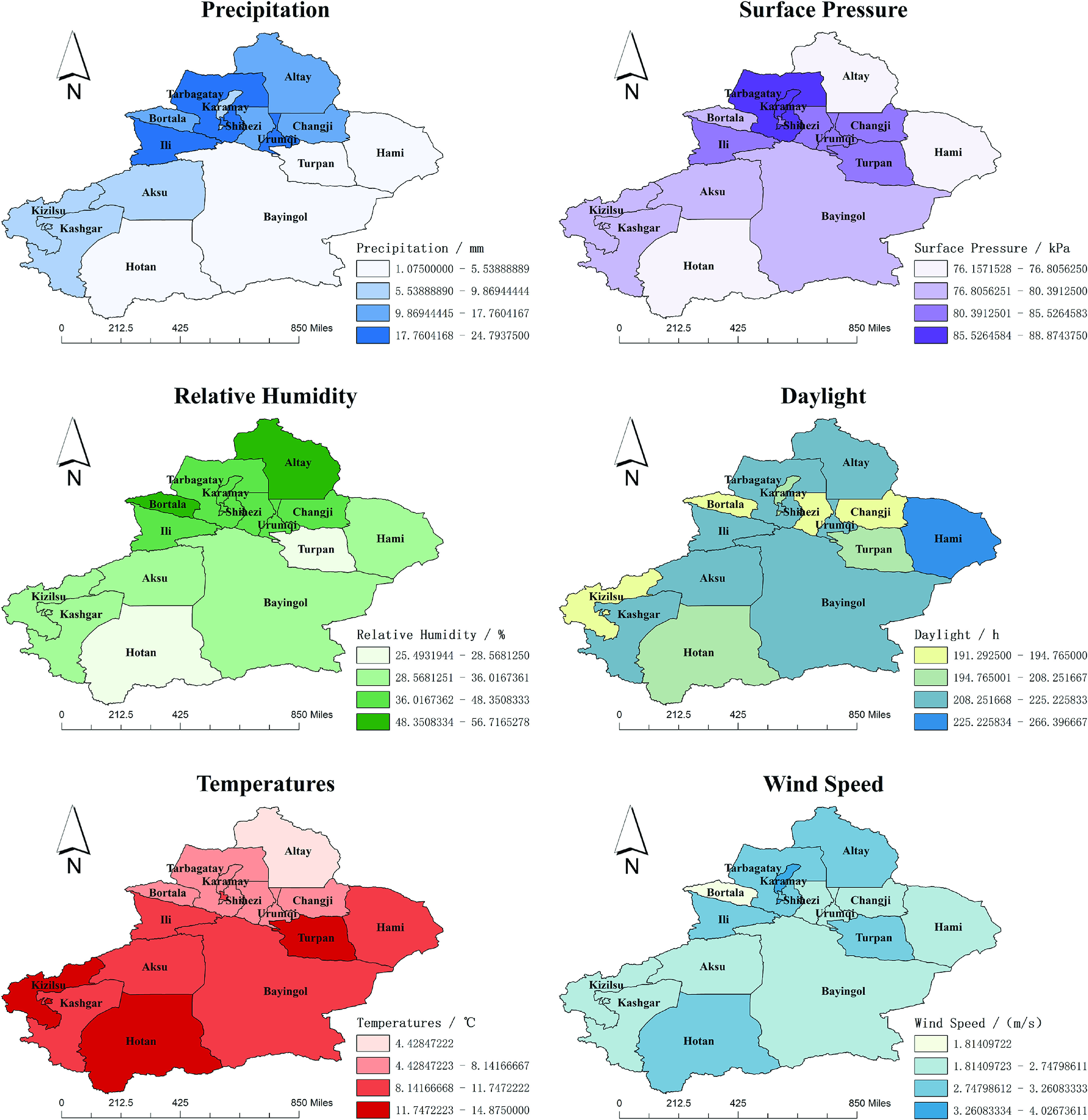
Monthly average values of meteorological indicators in Xinjiang regions.

The monthly means ± standard error of the mean (SEM) of precipitation, temperature, sunshine hours, relative humidity, wind speed, and surface pressure of Xinjiang in 2008–2018 were (13.88 ± 16.13) mm, (10.33 ± 13.41)°C, (233.55 ± 80.83) h, (43.18 ± 16.01)%, (3.03 ± 0.73) m/s, and (89.73 ± 4.68) kPa, respectively. Figure 3 reflects each region’s monthly mean distribution of the above factors. We observe that there are regional variations of each meteorological factor. We use Moran’s 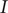 value to analyze the spatial autocorrelation of various meteorological factors in relation to HFMD incidence in Xinjiang from 2008 to 2018. The weight matrix of Moran’s 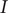 is generated by using the inverse distance weighting method. According to Table 1, Moran’s 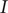 values are all greater than 0. This indicates that the incidence of HFMD is spatially positively correlated in Xinjiang during the period 2008–2018. The 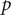 values are all less than 0.05 and statistically significant.

**Table 1.** Spatial autocorrelation analysis of HFMD in Xinjiang from 2008 to 2018

Year	2008	2009	2010	2011	2012	2013	2014	2015	2016	2017	2018
Moran’s 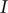	0.32	0.50	0.43	0.60	0.33	0.40	0.40	0.33	0.44	0.32	0.50
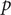 -value	0.04	0.01	0.02	0.01	0.02	0.02	0.01	0.02	0.02	0.04	0.01

The GTWR model is designed to analyze the influence of meteorological factors on HFMD transmission in time and space. The regression model is based on a weight matrix that integrates both temporal and spatial information [29]. Let 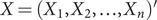 , where 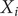 is the number of HFMD incidences in the 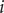 th region at time 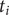 , and let 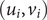 be the latitude and longitude coordinates of the 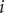 th region. The GTWR model is as follows:
(1)

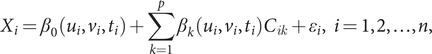

where 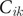 is the value of the 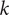 th meteorological variable in the 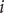 th region at time 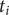 , 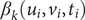 is the corresponding weight, 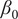 is the constant term and 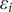 is the error term.

The coefficients 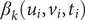 of meteorological factors can reflect the relationship between the HFMD incidence and meteorological variables. Through the weighted least square method and local linear geographical weighted regression, the estimates of the weight of the meteorological variables can be expressed as
(2)

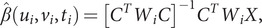

where 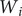 is the spatio-temporal weight matrix defined by spatio-temporal distance and bandwidth, and the elements in 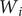 are generated by
(3)

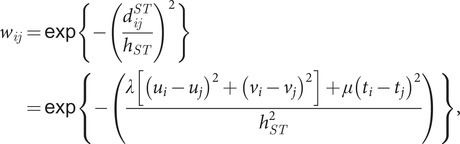

where 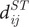 is the spatio-temporal distance between samples 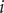 and 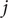 , 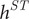 is the spatio-temporal bandwidth, 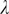 , and 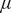 are scaling factors to determine the effects of spatial and temporal distances on the weights. After standardizing the HFMD data, Table 2 presents the average estimated values of the regression coefficients 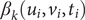 .

**Table 2.** The mean values of GTWR standardized coefficients of each meteorological variable

Region	Precipitation	Temperature	Surface pressure	Relative humidity	Wind speed	Daylight
Aksu	0.07	0.58	0.29	0.33	0.34	0.60
Altay	0.17	0.46	0.04	0.62	0.41	0.60
Bayingol	0.06	0.59	0.32	0.33	0.36	0.60
Bortala	0.18	0.50	0.30	0.58	0.17	0.52
Changji	0.17	0.51	0.67	0.43	0.38	0.52
Hami	0.03	0.56	0.08	0.27	0.36	0.72
Hotan	0.04	0.62	0.06	0.19	0.44	0.54
Ili	0.24	0.55	0.56	0.41	0.48	0.59
Karamay	0.10	0.53	0.90	0.41	0.69	0.54
Kashgar	0.08	0.60	0.26	0.29	0.32	0.60
Kizilsu	0.09	0.61	0.26	0.29	0.32	0.51
Shihezi	0.18	0.52	0.87	0.44	0.39	0.54
Tarbagatay	0.25	0.52	0.80	0.51	0.51	0.58
Turpan	0.01	0.65	0.67	0.23	0.43	0.56
Urumqi	0.24	0.52	0.67	0.43	0.38	0.59

According to Table 2, the coefficients of precipitation are generally smaller than those of all factors. Therefore, this factor does not significantly affect the transmission of HFMD in each region. The effects of temperature and daylight are substantial in most regions. For different regions, other factors influencing the spread of HFMD vary significantly. For example, surface pressure is the most influential factor in Changji, Urumqi, and Tarbagatay, while it is insignificant in Hami and Hotan. Thus, these meteorological factors have significant spatial heterogeneity. The spread of HFMD transmission cannot be accurately described if meteorological factors are treated equally across all regions. Based on the above analysis, we conduct variable selection using the GTWR model. Then, the variables that have a significant impact on each region are incorporated into the prediction model.

## The SARIMA-XGBoost model

In this section, we propose a combined model for investigating HFMD in Xinjiang, which integrates the SARIMA model and the XGBoost algorithm. While the SARIMA model analyzes seasonal disease trends, the XGBoost algorithm addresses the nonlinear influence of meteorological factors.

### SARIMA model

The SARIMA model can transform a non-stationary time series into stationary time ones. It is effective for studying time series with seasonal trends. To maintain the stationarity of the series, the trend and seasonality of HFMD incidence are eliminated using differencing [30]. Let 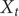 be the confirmed cases of HFMD at time 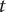 , and 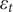 is the error term. A SARIMA model is defined as
(4)

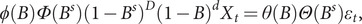

where 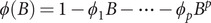 and 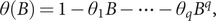 corresponding to the functions of the backshift operator 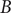 with 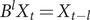 . Here, 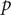 is the autoregressive order, 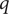 is the moving average order, and 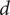 is the number of differencing operations. To eliminate seasonal variations, the SARIMA model uses seasonal differentials 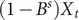 , where 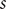 is the seasonal period of the data. The forms of 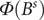 and 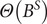 are as shown below:

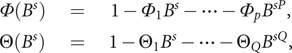

where 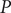 is the seasonal autoregressive order, 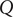 is the seasonal moving average order, and 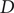 is the number of seasonal differencing operations. We call (4) an SARIMA 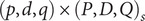 model.

After removing the trend and seasonal components, the model fitting process includes order determination, parameter estimation, and diagnostic validation. The range of orders is determined by the autocorrelation function (ACF) and partial autocorrelation function (PACF). Within this range, multiple order combinations are traversed to obtain the optimal parameters that minimize the Akaike information criterion (AIC) and Bayesian information criterion (BIC) [31]. The parameters of the model are then estimated. In the diagnostic validation phase, the residuals are tested for normality and autocorrelation using the Shapiro–Wilk and Ljung–Box tests. The Shapiro–Wilk test, proposed by [32], is a normality test as follows:
(5)

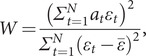

where 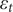 is the residual of the SARIMA model, 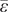 is the sample mean, and the coefficient 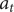 is the expected value of the standard normal statistic. Ljung–Box test [33, 34] is expressed as
(6)

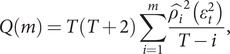

where 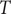 is the sample size, i.e. the number of months included in the dataset, 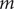 is the maximum lagging order, 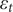 is the residuals of the SARIMA model, and 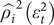 is the 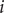 th order of the sample ACF. The Ljung–Box test reflects the autocorrelation of the series based on the autocorrelation coefficient of the series lagged at order 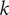 .

### XGBoost algorithm

The XGBoost algorithm is an ensemble learning algorithm that incorporates a regularization term to control model complexity and avoid overfitting. Based on the classification and regression tree algorithm [35], XGBoost is constructed by iteratively fitting the negative gradient values of the loss function to form a new model. As a result, it performs better in analyzing nonlinear data [36]. Next, we review some basic concepts of the XGBoost algorithm [37]. Given 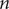 observations 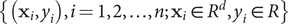 , a tree ensemble model is established to predict the output where 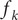 is the kth decision tree, satisfying 
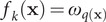
 for 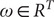 and 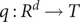 , 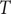 is the number of leaf nodes in the tree, and 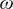 is the weight vector of leaf nodes. The regularized objective function 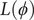 is composed of a loss function 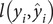 and a regular term 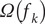 as follows:

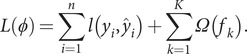

The second term 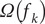 penalizes the complexity of regression three functions and is defined by 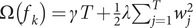 , where 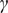 is the coefficient of the number of leaf nodes. Let 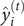 be the prediction of the 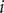 th iteration. Since XGBoost uses the gradient-boosting decision tree pattern for the training set, it follows that 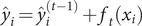 . Thus, we need to minimize the following objectives:

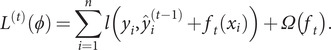



On the other hand, a second-order Taylor expansion at 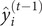 is derived from the loss function. Therefore, the loss function is rewritten as follows:

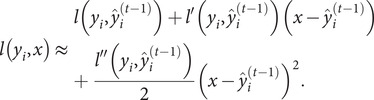



Denote 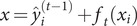 , 
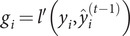
 and 
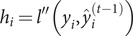
 . The following objective can approximate the above objective function:
(7)

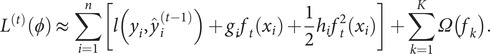



### The combined SARIMA-XGBoost model

Through the analysis above, the SARIMA model can capture the historical patterns of HFMD prevalence well but cannot account for the endogenous factors affecting prevalence or capturing the complex factors of transmission due to its linear assumptions. To more accurately investigate the trend of HFMD cases, we introduce the XGBoost algorithm to effectively capture the nonlinear characteristics [21]. We propose a combined SARIMA-XGBoost model to analyze both the linear and nonlinear components of the HFMD series. First, the SARIMA model is used to analyze the linear part of the series. Then, the residuals of the SARIMA model are considered the nonlinear part and are analyzed using the XGBoost model. Ignoring the effect of specific nonlinear factors can lead to poor performance in some situations. To address this, meteorological variables such as wind speed, relative humidity, and surface air pressure are included in the input layer of the XGBoost algorithm.

**Figure 4.**
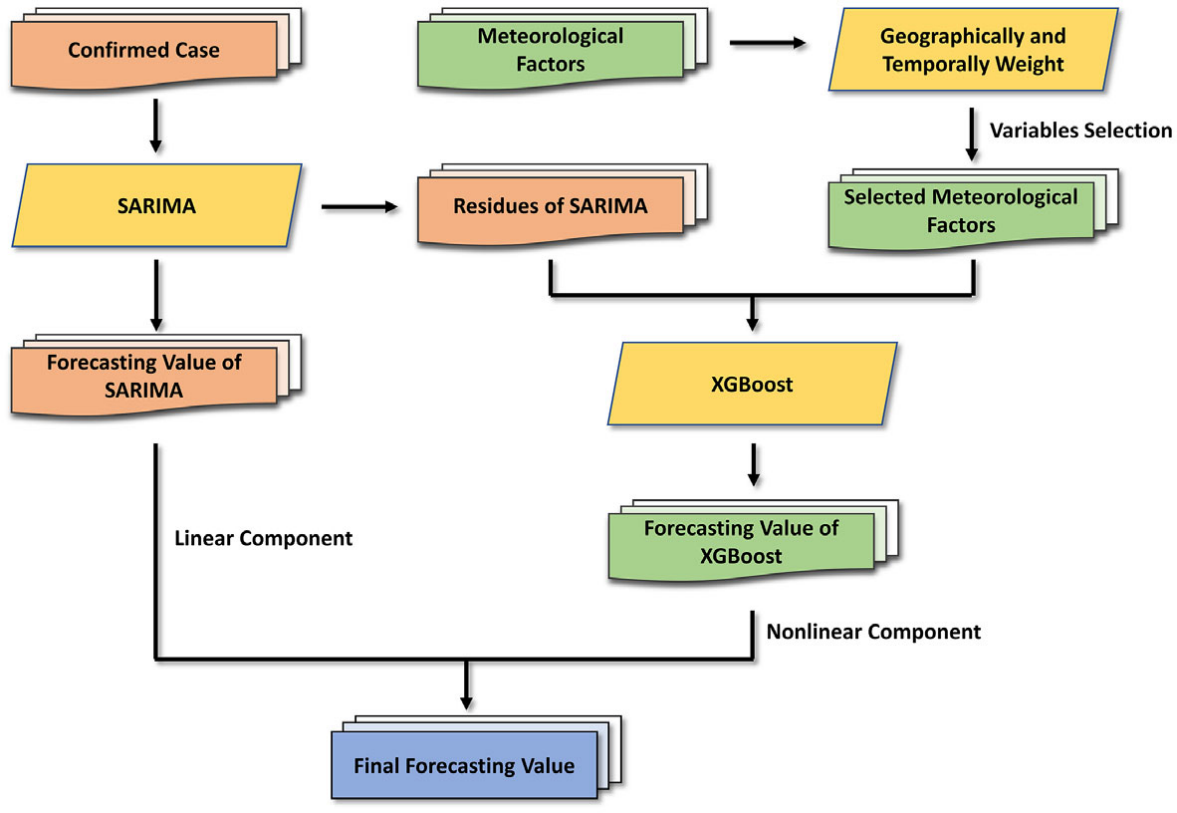
The flow chart of the combined SARIMA-XGBoost model.

The flow chart of the SARIMA-XGBoost model is shown in Figure 4. Let 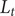 and 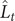 be the true and fitting values from the SARIMA model (4) at time 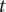 , respectively. Suppose that the HFMD series is formed by the linear and nonlinear components:

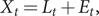

where 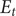 denotes the nonlinear part based on the XGBoost algorithm (7). The detailed process of the model is described as follows:

- Through the SARIMA model (4), we obtain the corresponding residual values denoted by
(8)

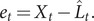

- Analyze the meteorological factors for each region based on the GTWR model (1) and select the influential factors as meteorological variables denoted by 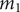 , 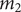 , 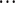 , 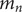 .
- Apply the XGBoost algorithm (7) to model the residuals (8). With *n* meteorological variables, the residual model is established as
(9)

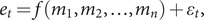

where 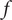 is a nonlinear function determined by the XGBoost algorithm, and 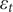 is the random error. Let 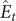 be the estimated values of 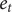 . The estimator 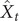 of 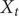 *is*


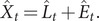



To ensure a comprehensive and balanced evaluation, we employ four indexes to assess the performance of the combined SARIMA-XGBoost model: the root mean square error (RMSE), coefficient of determination ( 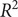 ), mean absolute error (MAE), and symmetric mean absolute percentage error (SMAPE). RMSE offers insights into the forecast’s accuracy by quantifying the average discrepancies between the actual and predicted values. 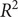 , a measure of the model’s goodness of fit, indicates the percentage of variance in the dependent variable accounted for by the independent variables. MAE measures the mean magnitude of errors by averaging the absolute differences between observed and predicted values. In addition, SMAPE provides a relative error measurement, factoring in symmetric penalties for both overpredictions and underpredictions. The model’s accuracy is considered higher as 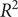 approaches 1 and as RMSE, MAE, and SMAPE values decrease. The formulas to compute these metrics are as follows:

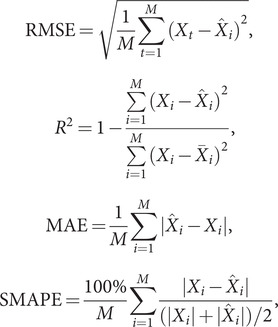

where 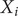 is the actual observed value, 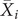 is the mean value of 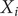 , 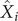 is the predicted value, and 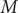 denotes the number of samples.

## Experiment and analysis

### Analysis of SARIMA-XGBoost model

In the SARIMA-XGBoost model, we first establish the SARIMA model determined by the following steps:

- Apply the augmented Dickey–Fuller test to determine the stationarity of the HFMD incidence series in 15 regions of Xinjiang from 2008 to 2018. If the series is non-stationary, use differencing to transform it into a stationary series.
- Determine six parameters 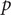 , 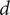 , 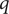 , 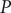 , 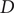 , and 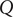 according to the ACF and PACF plots.
- Based on the AIC and BIC criteria, conduct multiple order combinations to determine the optimal parameters.

The model residuals are analyzed through the Shapiro–Wilk and Ljung–Box tests to determine the effectiveness of the SARIMA model. The diagnosis of the model and the test results are shown in Table 3. All the 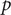 values exceed the significance level of 0.05, indicating that the residual series pass the Ljung–Box test. This suggests that the SARIMA model successfully captures the temporal autocorrelation in the HFMD incidence data. However, all 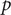 values of the Shapiro–Wilk test are less than 0.05, the residual series do not pass the normality test. This may indicate that the SARIMA model has not adequately captured the structure of the data, and there could be nonlinear trends present. Using the SARIMA model alone is less effective for analyzing HFMD trends in each region.

**Table 3.** Diagnosis of SARIMA (*p, d, q*) × (*P, D, Q*)*_s_* model

Region	Model	AIC	BIC	Shapiro–Wilk	Ljung–Box Q(6)	Ljung–Box Q(12)	Ljung–Box Q(24)
Aksu	SARIMA(1, 0, 1) × (0, 1, 1)_12_	825	835	0.66(5.03E-16)	4.27(0.64)	17.40(0.14)	18.55(0.78)
Altay	SARIMA(1, 0, 1) × (0, 1, 1)_12_	913	923	0.78(1.02E-12)	4.13(0.66)	10.32(0.59)	19.18(0.74)
Bayingol	SARIMA(0, 0, 1) × (1, 1, 1)_12_	1090	1101	0.57(5.88E-18)	8.40(0.21)	11.87(0.37)	8.51(0.58)
Bortala	SARIMA(1, 0, 1) × (0, 1, 1)_12_	825	835	0.76(1.71E-13)	2.47(0.87)	4.13(0.98)	8.56(1.00)
Changji	SARIMA(1, 0, 1) × (1, 1, 1)_12_	1133	1146	0.85(2.50E-10)	5.12(0.53)	14.29(0.28)	20.49(0.67)
Hami	SARIMA(0, 0, 1) × (0, 1, 1)_12_	1033	1041	0.48(1.13E-19)	1.10(0.98)	5.47(0.94)	20.87(0.65)
Hotan	SARIMA(0, 0, 1) × (0, 1, 1)_12_	532	540	0.53(1.04E-18)	1.48(0.96)	4.75(0.97)	11.04(0.99)
Ili	SARIMA(1, 1, 1) × (1, 1, 1)_12_	1277	1290	0.80(2.69E-12)	4.64(0.59)	9.18(0.69)	32.72(0.11)
Kashgar	SARIMA(0, 0, 1) × (0, 1, 1)_12_	617	625	0.67(9.31E-16)	1.81(0.94)	8.46(0.75)	12.03(0.98)
Karamay	SARIMA(1, 0, 1) × (1, 1, 1)_12_	1029	1039	0.69(2.08E-15)	2.48(0.87)	11.22(0.51)	24.40(0.44)
Kizilsu	SARIMA(1, 1, 1) × (1, 1, 1)_12_	454	467	0.87(1.33E-09)	2.58(0.86)	5.10(0.95)	7.89(1.00)
Shihezi	SARIMA(1, 0, 1) × (0, 1, 1)_12_	1069	1080	0.45(3.15E-20)	0.89(0.99)	2.01(1.00)	11.66(0.98)
Tarbagatay	SARIMA(1, 1, 1) × (0, 1, 1)_12_	1106	1116	0.79(1.58E-12)	5.44(0.49)	11.74(0.47)	23.18(0.51)
Turpan	SARIMA(1, 0, 1) × (1, 1, 1)_12_	1084	1097	0.81(6.17E-12)	0.33(0.99)	2.43(0.99)	8.72(0.99)
Urumqi	SARIMA(1, 0, 1) × (1, 1, 1)_12_	1330	1343	0.53(8.03E-19)	1.29(0.97)	4.00(0.98)	9.68(1.00)

To capture the nonlinear characteristics in the residuals, based on the SARIMA (*p, d, q*) × (*P, D, Q*)*_s_* model listed in Table 3, meteorological variables corresponding to each region serve as input for the XGBoost algorithm, as introduced in equation (7). During the training of the XGBoost algorithm, the choice of parameters significantly affects the model’s effectiveness. Thus, parameters like the maximum number of iterations, maximum tree depth, and random sampling ratio are carefully considered. For instance, in Urumqi, the chosen parameters are a maximum of 4000 iterations, a maximum tree depth of 6, a learning rate of 0.1, and a random sampling ratio of 0.5. Details on the parameter-tuning process will follow:

- Considering the different factors, we first normalize the meteorological data. Based on the standardized regression coefficient (Table 2) of the GTWR model, the four important factors are selected as input variables of the XGBoost algorithm for each region.
- Determine the ‘booster’ to be ‘gbtree’; that is, the tree model is regarded as the base model. The objective parameter is selected as ‘reg:squarederror’, corresponding to a regression problem with minimizing MSE.
- Take the learning rate to be 0.1, and then use the built-in ‘xgb.csv’ function. The ‘xgb.csv’ function will return the optimal maximum number of iterations.
- For the remaining parameters, the range of parameters is first determined, and then the ‘GridSearchCV’ function is used to traverse and search the optimal parameters.

In the implementation of the XGBoost algorithm, a tree model is used as the base model. Within this tree model, input variables serve as split nodes and are associated with different gain values of the objective function. A higher gain value indicates a greater influence of the variable on the model. Therefore, the importance of each variable can be assessed by the frequency with which it is used as a split node. Table 4 presents the feature importance of the meteorological variables, measured by their use as split nodes during the training of the XGBoost algorithm. This provides insights into the impact of the selected meteorological factors on the incidence of HFMD in each region.

**Table 4.** The feature important and percentage of meteorological variables for each region

Region	Temperature	Surface pressure	Relative humidity	Wind speed	Daylight
Aksu	3351(0.27)	–	3052(0.24)	2772(0.22)	3310(0.27)
Altay	3164(0.23)	–	3883(0.28)	3028(0.22)	3640(0.27)
Bayingol	4175(0.26)	–	4030(0.25)	3627(0.23)	4254(0.26)
Bortala	3353(0.25)	3556(0.26)	3480(0.26)	–	3232(0.24)
Changji	4619(0.26)	4162(0.24)	4193(0.24)	–	4458(0.26)
Hami	3288(0.24)	–	3235(0.23)	4126(0.3)	3155(0.23)
Hotan	550(0.25)	–	583(0.26)	493(0.22)	583(0.26)
Ili	4195(0.25)	3915(0.24)	–	3550(0.21)	4992(0.3)
Karamay	3214(0.23)	3736(0.27)	–	3640(0.26)	3435(0.24)
Kashgar	1546(0.27)	–	1455(0.25)	1427(0.25)	1290(0.23)
Kizilsu	380(0.32)	–	275(0.23)	190(0.16)	333(0.28)
Shihezi	4020(0.25)	3927(0.24)	3960(0.25)	–	4145(0.26)
Tarbagatay	3731(0.25)	4271(0.29)	–	3560(0.24)	3264(0.22)
Turpan	3999(0.26)	4355(0.28)	–	3526(0.23)	3711(0.24)
Urumqi	4577(0.27)	4185(0.24)	4213(0.25)	–	4160(0.24)

### Compared with other models

In this section, we compare the performance of the SARIMA-XGBoost model to other models, such as SARIMA, XGBoost, long-/short-term memory (LSTM) networks, and support vector regression (SVR) models. The process for establishing the SARIMA-XGBoost model is described in the Section ‘The SARIMA-XGBoost model’. To provide effective information for the rational allocation of medical resources across various regions, we use a dataset comprising the number of HFMD patients in 15 regions of Xinjiang from 2008 to 2018. The first nine years of data serve as the training set, while the data from 2017 to 2018 is used as the test set. The regional incidence can also be predicted by dividing the number of patients by the total number of people in the area, which will help eliminate the effect of differences in population density and make comparisons between different regions fairer.

**Figure 5.**
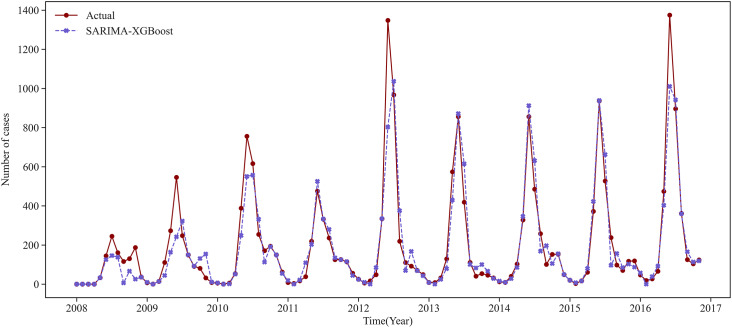
The fitting result graph for the training set in Urumqi.

**Figure 6.**
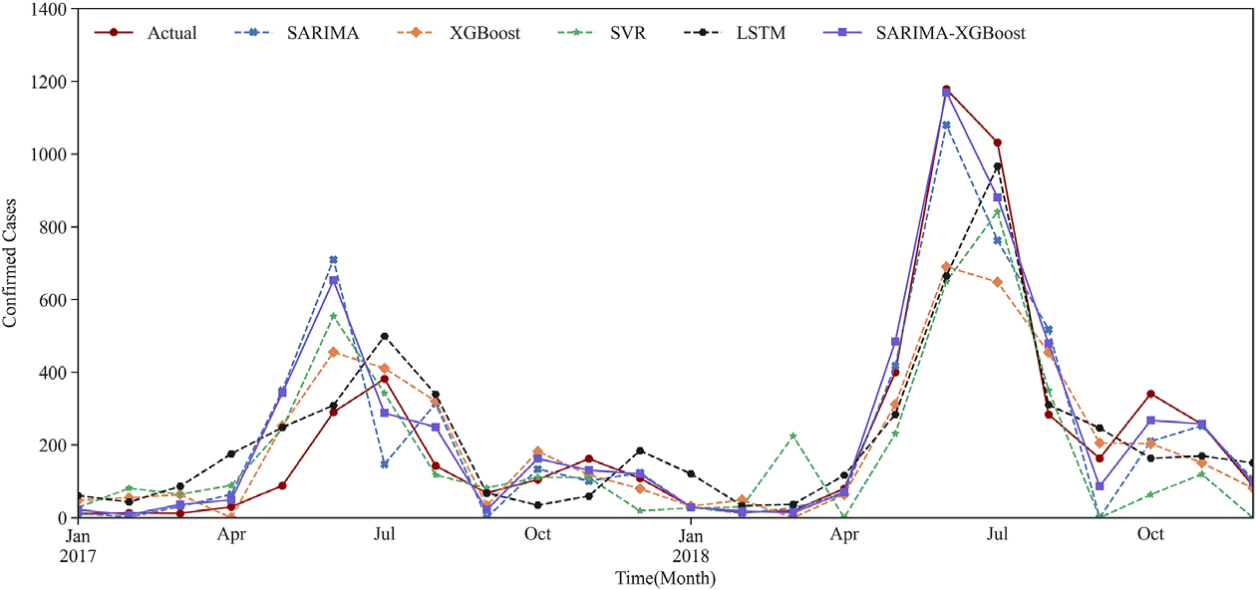
The prediction results of Urumqi in five models.

We use the prediction results for Urumqi as an example. Based on the critical meteorological factors outlined in Table 4, Figures 5 and 6 show the fitting result graph for the training set and the comparison of prediction in Urumqi. When comparing with other models, we individually optimize each model to ensure predictive accuracy. For example, given the XGBoost model’s strength in ensemble learning, we incorporate meteorological variables into its predictions. Numerical simulations show that the predictive accuracy of the LSTM model decreases when incorporating meteorological factors as input variables. Consequently, we use just the historical HFMD cases as the input feature for the LSTM model. It can be observed that the SARIMA-XGBoost model outperforms the other models. Specifically, the RMSE values for SARIMA, XGBoost, LSTM, and SVR are 147.51, 152.75, 129.43, and 167.01, respectively. The proposed SARIMA-XGBoost model has an RMSE of 112.51, which is significantly lower than those of the other models. The 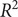 value for the SARIMA-XGBoost model is higher by 13.3%, 16.4%, 7.5%, and 25% when compared to SARIMA, XGBoost, LSTM, and SVR, respectively. Additionally, the SARIMA-XGBoost model exhibits the lowest values among the five models for both MAE and SMAPE metrics.

**Figure 7.**
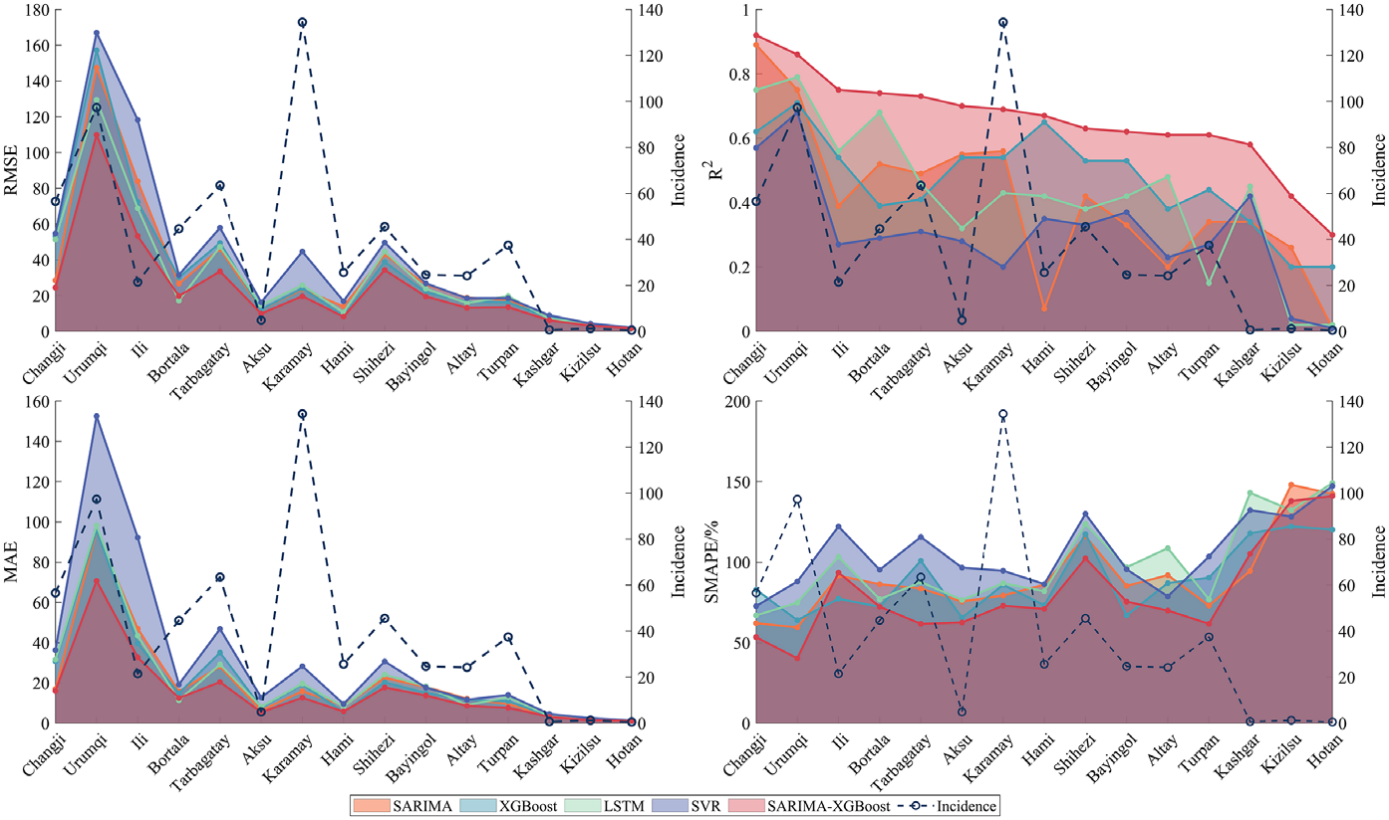
Evaluation results of different models for the prediction of 15 regions in Xinjiang.

Figure 7 presents the RMSE, 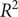 , MAE, and SMAPE values of five models used for predicting the 15 regions in Xinjiang during 2017–2018. Considering the above four metrics, the SARIMA-XGBoost model significantly outperforms other models in prediction. For example, the 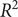 of the SARIMA-XGBoost model increases by 58.5%, 38.3%, 54%, and 111%, compared to the SARIMA model, XGBoost algorithm, LSTM, and SVR model, respectively. In regions with higher incidence, such as Changji, Urumqi, Bortala, Tarbagatay, and Karamay, the SARIMA-XGBoost model demonstrates minor deviations and greater robustness than other models, with a notable improvement in accuracy. However, for regions with lower incidence like Kizilsu, Kashgar, and Hotan, the prediction accuracy of the SARIMA-XGBoost model is comparatively lower. One of the primary reasons is that both SARIMA models and machine learning methods demand ample data to achieve accurate predictions, which is lacking in these regions with lower incidence. For instance, over the past decade in Hotan, around 73.5% of the months reported zero cases, and approximately 93.2% of the months reported fewer than five cases. This limited data poses challenges for precise predictions. Moreover, the differences and fluctuations of meteorological factors in regions with low incidence are not as pronounced. Despite these challenges, the SARIMA-XGBoost model still significantly enhances RMSE, 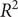 , MAE, and SMAPE values compared with other models.

Based on the above analysis, we can conclude that the proposed SARIMA-XGBoost model can achieve better performance in predicting HFMD.

## Discussion and conclusion

In this paper, we propose a hybrid method based on the combination of the SARIMA model and XGBoost algorithm to improve the prediction accuracy of the HFMD time series. The SARIMA model can capture typical trends and seasonal characteristics of HFMD, and the XGBoost analyzes the influence of meteorological factors. Since precipitation, temperature, and relative humidity have different effects for each region, the GTWR model is designed to investigate the impact of meteorological factors. The prediction and verification experiment of Xinjiang HFMD incidence data shows that the proposed SARIMA-XGBoost model is superior to other models in accuracy, especially in regions with high incidence. Based on the SARIMA-XGBoost model, we derive several conclusions of practical significance: (i) The HFMD exhibits seasonal characteristics, peaking from May to August each year; (ii) The HFMD incidence has significant geographical aggregation. It is highly prevalent in the northern regions of Xinjiang, such as Urumqi and Karamay. The incidence is relatively low in southern Xinjiang, such as Hotan, Kashgar, Kizilsu, and Aksu; and (iii) The meteorological factors exhibit significant spatial heterogeneity. For instance, surface pressure is a dominant factor in Changji, Urumqi, and Tarbagatay, but it holds little significance in Hami and Hotan.

Besides the HFMD series, we observe that many time series of other diseases, such as influenza and malaria, are also influenced by linear and nonlinear factors. The hybrid method of combining the time series model with machine learning algorithms is of great significance in fully extracting the information and improving forecasting accuracy. Therefore, how to select the appropriate model and design the combining method needs to be considered in the future.

## Data Availability

The data on HFMD cases between 2008 and 2018 are from [10]. The meteorological data of monthly average temperature, average precipitation, barometric pressure, sunshine hours, average humidity, and wind speed are from NASA (https://ladsweb.modaps.eosdis.nasa.gov). The datasets and software code used during this current study are available from the corresponding authors at a reasonable request.

## References

[1] World Health Organization (2011) A Guide to Clinical Management and Public Health Response for Hand, Foot and Mouth Disease (HFMD). Available at https://www.who.int/Westernpacific.

[2] Ventarola D, Bordone L and Silverberg N (2015) Update on hand-foot-and-mouth disease. Clinics in Dermatology 33, 340–346. 25889136 10.1016/j.clindermatol.2014.12.011

[3] Chan LG, Parashar UD, Lye MS, Ong FGL, Zaki SR, Alexander JP, Ho KK, Han LL, Pallansch MA, Suleiman AB, Jegathesan M, Anderson LJ and Outbreak Study Group (2000) Deaths of children during an outbreak of hand, foot, and mouth disease in Sarawak, Malaysia: Clinical and pathological characteristics of the disease. Clinical Infectious Diseases 31, 678–683. 11017815 10.1086/314032

[4] Xie L, Huang R, Wang H and Liu S (2021) Spatial-temporal heterogeneity and meteorological factors of hand-foot-and-mouth disease in Xinjiang, China from 2008 to 2016. PLoS One 16, e0255222. 34339424 10.1371/journal.pone.0255222 PMC8328314

[5] Lafferty KD (2009) The ecology of climate change and infectious diseases. Ecology 90, 888–900. 19449681 10.1890/08-0079.1

[6] Epstein PR (2001) Climate change and emerging infectious diseases. Microbes and Infection 3, 747–754. 11489423 10.1016/s1286-4579(01)01429-0

[7] World Health Organization (2005) Using Climate to Predict Infectious Disease Epidemics. Available at https://www.who.int/Westernpacific.

[8] Onozuka D and Hashizume M (2011) The influence of temperature and humidity on the incidence of hand, foot, and mouth disease in Japan. Science of the Total Environment 410, 119–125. 22014509 10.1016/j.scitotenv.2011.09.055

[9] Hii YL, Rocklöv J and Ng N (2011) Short term effects of weather on hand, foot and mouth disease. PloS One 6, e16796. 21347303 10.1371/journal.pone.0016796 PMC3037951

[10] Sun S, Li Z, Hu X and Huang R (2021) Spatiotemporal characters and influence factors of hand, foot and mouth epidemic in Xinjiang, China. PloS One 16, e0254223. 34428212 10.1371/journal.pone.0254223 PMC8384200

[11] Ma E, Lam T, Wong C and Chuang SK (2010) Is hand, foot and mouth disease associated with meteorological parameters? Epidemiology and Infection 138, 1779–1788. 20875200 10.1017/S0950268810002256

[12] Hong ZM, Wang HH, Wang YJ and Wang WR (2020) Spatiotemporal analysis of hand, foot and mouth disease data using time-lag geographically- weighted regression. Geospatial Health 15, 849. 10.4081/gh.2020.849 33461279

[13] Yi S, Wang H, Yang S, Xie L, Gao Y and Ma C (2021) Spatial and temporal characteristics of hand-foot-and-mouth disease and its response to climate factors in the Ili River valley region of China. International Journal of Environmental Research and Public Health 18, 1954. 33671423 10.3390/ijerph18041954 PMC7923010

[14] Koh WM, Bogich T, Siegel K, Jin J, Chong EY, Tan CY, Chen MIC, Horby P and Cook AR (2016) The epidemiology of hand, foot and mouth disease in Asia: A systematic review and analysis. The Pediatric Infectious Disease Journal 35, 285. 10.1097/INF.0000000000001242 PMC5130063 27273688

[15] Chen KT, Chang HL, Wang ST, Cheng YT and Yang JY (2007) Epidemiologic features of hand-foot-mouth disease and herpangina caused by enterovirus 71 in Taiwan, 1998–2005. Pediatrics 120, 244–252. 17671037 10.1542/peds.2006-3331

[16] Zhang J, Sun J, Chang Z, Zhang W, Wang Z and Feng Z (2011) Characterization of hand, foot, and mouth disease in China between 2008 and 2009. Biomedical and Environmental Sciences 24, 214–221. 21784305 10.3967/0895-3988.2011.03.002

[17] Liu L, Luan RS, Yin F, Zhu XP and Lü Q (2016) Predicting the incidence of hand, foot and mouth disease in Sichuan province, China using the ARIMA model. Epidemiology and Infection 144, 144–151. 26027606 10.1017/S0950268815001144 PMC9507307

[18] Tian C, Wang H and Luo X (2019) Time-series modelling and forecasting of hand, foot and mouth disease cases in China from 2008 to 2018. Epidemiology and Infection 147, e82. 30868999 10.1017/S095026881800362X PMC6518604

[19] Liu S, Chen J, Wang J, Wu Z, Wu W, Xu Z, Hu W, Xu F, Tong S and Shen H (2018) Predicting the outbreak of hand, foot, and mouth disease in Nanjing, China: A time-series model based on weather variability. International Journal of Biometeorology 62, 565–574. 29086082 10.1007/s00484-017-1465-3

[20] Sioofy Khoojine A, Shadabfar M, Hosseini VR and Kordestani H (2021) Network autoregressive model for the prediction of COVID-19 considering the disease interaction in neighboring countries. Entropy 23, 1267. 34681991 10.3390/e23101267 PMC8535150

[21] Zhang G (2003) Time series forecasting using a hybrid ARIMA and neural network model. Neurocomputing 50, 159–175.

[22] Liao J, Yu S, Yang F, Yang M, Hu Y and Zhang J (2016) Short-term effects of climatic variables on hand, foot, and mouth disease in mainland China, 2008–2013: A multilevel spatial Poisson regression model accounting for overdispersion. PLoS One 11, e0147054. 26808311 10.1371/journal.pone.0147054 PMC4726563

[23] Ma T, Ji T, Yang G, Chen Y, Xu W and Liu H (2021) Incidence trend prediction of hand-foot-mouth disease based on long short-term memory neural network. Journal of Computer Applications 41, 265.

[24] Wang Y, Xu C, Zhang S, Yang L, Wang Z, Zhu Y and Yuan J (2019) Development and evaluation of a deep learning approach for modeling seasonality and trends in hand-foot-mouth disease incidence in mainland China. Scientific Reports 9, 1–15. 31142826 10.1038/s41598-019-44469-9 PMC6541597

[25] Nguyen T and Minh D (2021) Applying machine learning to predict hand-foot-mouth disease outbreaks in Vietnam. Journal of Health Informatics in Developing Countries 15(2).

[26] Lin X, Wang X, Wang Y, Du X, Jin L, Wan M, Ge H and Yang X (2021) Optimized neural network based on genetic algorithm to construct hand-foot-and-mouth disease prediction and early-warning model. International Journal of Environmental Research and Public Health 18, 2959. 33799332 10.3390/ijerph18062959 PMC8001304

[27] Liu LL, Hu YC, Qi C, Zhu YC, Li CY, Wang L, Cui F and Li XJ (2022) Comparison of different predictive models on HFMD based on weather factors in Zibo city, Shandong Province, China. Epidemiology and Infection 150, e10.

[28] Dietterich T (1995) Overfitting and undercomputing in machine learning. ACM Computing Surveys (CSUR) 27, 326–327.

[29] Huang B, Wu B and Barry M (2010) Geographically and temporally weighted regression for modeling spatio-temporal variation in house prices. International Journal of Geographical Information Science 24, 383–401.

[30] Box GE, Jenkins JM, Reinsel GC and Ljung GM (2015) Time Series Analysis: Forecasting and Control. Hoboken, NJ: John Wiley & Sons.

[31] Burnham KP and Anderson DR. (2004) Multimodel inference: Understanding AIC and BIC in model selection. Sociological Methods & Research 33, 261–304.

[32] Shapiro SS and Wilk MB (1965) An analysis of variance test for normality (complete samples). Biometrika 52, 591–611.

[33] Box GEP and Pierce DA (1970) Distribution of residual autocorrelations in autoregressive-integrated moving average time series models. Journal of the American Statistical Association 65, 1509–1526.

[34] Ljung GM and Box GEP (1978) On a measure of lack of fit in time series models. Biometrika 65, 297–303.

[35] De’ath G and Fabricius KE (2000) Classification and regression trees: A powerful yet simple technique for ecological data analysis. Ecology 81, 3178–3192.

[36] Ridgeway G (1999) The state of boosting. Computing Science and Statistics 172–181.

[37] Chen T and Guestrin C (2016) Xgboost: A scalable tree boosting system. In Proceedings of the Proceedings of the 22nd ACM SIGKDD International Conference on Knowledge Discovery and Data Mining, pp. 785–794.

